# Supplementary material for: Causal Relationship between Aging and Anorexia Nervosa: A White-Matter-Microstructure-Mediated Mendelian Randomization Analysis
Source: Biomedicines. 2024 Aug 16;12(8):1874. doi: 10.3390/biomedicines12081874 (PMC11351342; doi:10.3390/biomedicines12081874)
Supplement: Supplementary file 1 [file biomedicines-12-01874-s001.zip › Supplementary Figure/Figure S8/Leaveoneout plot of MR analysis for TL to ubm-b-1513.pdf]

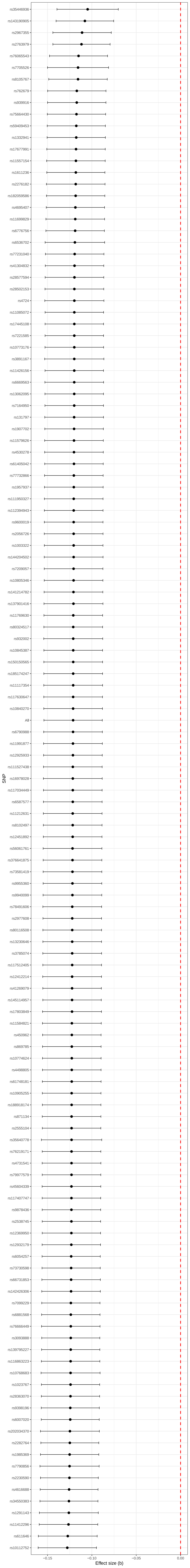

rs35446936
rs143190905
rs2967355
rs2763979
rs76065543
rs7705526
rs8105767
rs762679
rs939916
rs75664430
rs59409453
rs1332941
rs17677991
rs11557154
rs1611236
rs2276182
rs182059586
rs4695407
rs11699829
rs6776756
rs6536702
rs77231040
rs41304832
rs28577594
rs28502153
rs4724
rs11085072
rs17445108
rs7221585
rs10773176
rs3891167
rs11426156
rs6669563
rs13062095
rs7164950
rs131797
rs1907702
rs11579626
rs4530278
rs61405042
rs77732866
rs1957937
rs111950327
rs112394943
rs9600019
rs2056726
rs1003322
rs144204502
rs7209057
rs10805346
rs141214782
rs137901416
rs11769630
rs80324517
rs932002
rs10845387
rs150150565
rs185174247
rs11117354
rs117630647
rs10840270
All
rs6790988
rs11991877
rs12925933
rs111527438
rs16978028
rs117034449
rs6587577
rs11212631
rs8102497
rs12451892
rs56061761
rs376641875
rs73581419
rs9955360
rs9940099
rs78491606
rs2977608
rs80116508
rs13230646
rs3785074
rs117512405
rs12412214
rs41269079
rs145114957
rs17803849
rs11584821
rs450962
rs869785
rs10774624
rs4498805
rs61748181
rs10905255
rs188918174
rs871134
rs2555104
rs35640778
rs76219171
rs4731541
rs79977579
rs45604339
rs117407747
rs9878436
rs2538745
rs12369950
rs12932179
rs6054257
rs73730598
rs66731853
rs142426306
rs7099229
rs6881568
rs76666449
rs3093888
rs139795227
rs116863223
rs10768683
rs1023767
rs28363070
rs9398196
rs6007020
rs202034370
rs2282764
rs1985369
rs7790856
rs2230590
rs4616688
rs34550383
rs1291143
rs11412296
rs611646
rs10112752
SNP
−0.15
−0.10
−0.05
0.00
Effect size (b)
